# *Notes from the Field:* Cyclosporiasis Cases Associated with Dining at a Mediterranean-Style Restaurant Chain — Texas, 2017

**DOI:** 10.15585/mmwr.mm6721a5

**Published:** 2018-06-01

**Authors:** Amelia A. Keaton, Noemi Borsay Hall, Rebecca J. Chancey, Vivienne Heines, Venessa Cantu, Varsha Vakil, Stephen Long, Kirstin Short, Elya Franciscus, Natasha Wahab, Aisha Haynie, Laura Gieraltowski, Anne Straily

**Affiliations:** ^1^Division of Foodborne, Waterborne, and Environmental Diseases, National Center for Emerging and Zoonotic Infectious Diseases, CDC; ^2^Epidemic Intelligence Service, CDC; ^3^Texas Department of State Health Services; ^4^Division of Parasitic Diseases and Malaria, Center for Global Health, CDC; ^5^Bureau of Epidemiology, Houston Health Department, Texas; ^6^Harris County Health Department, Texas.

During July 21–August 8, 2017, the Texas Department of State Health Services (DSHS) was notified of 20 cases of cyclosporiasis among persons who dined at a Mediterranean-style restaurant chain (chain A) in the Houston area. On August 10, 2017, DSHS requested assistance from CDC to support ongoing investigations by the City of Houston Health Department, Harris County Public Health, Fort Bend County Health and Human Services, and Brazoria County Health Department. The objectives of this investigation were to determine the source of the illnesses in the Houston area and to generate hypotheses about the source of the national increase in cyclosporiasis in 2017.

Chain A has four locations in the Houston area and a central kitchen where many dishes are prepared. A case-control study was performed using a menu-specific questionnaire focusing on items containing fresh produce. A confirmed case was defined as laboratory-confirmed *Cyclospora* infection and clinically compatible illness in a person who ate at any location of chain A during May 28–July 15, 2017. A probable case was defined as diarrhea and at least one additional sign or symptom compatible with cyclosporiasis (e.g., anorexia, abdominal cramping, bloating, myalgia, fatigue, vomiting, or low-grade fever) in a person within 2 weeks after dining at chain A during May 28–July 15, 2017. Controls were identified as either dining companions of case-patients who had no illness or patrons who dined at the same chain A location within 2 days of a case-patient visit and who had no illness. For controls identified by the latter method, contact information was obtained using commercially available databases used by local health agencies in Texas. Three controls per case-patient were recruited.

A total of 22 case-patients (16 confirmed and six probable) and 66 controls were enrolled in the study. Case-patients had a median age of 52 years (range = 29–79 years); 50% were female. Analysis compared menu items consumed by case-patients and controls, followed by ingredient-level analysis. The following ingredients were identified as being significantly associated with illness: green onions (matched odds ratio = 11.3; 95% confidence interval = 2.55–104.68), tomatoes (5.5; 1.2–51.7), red onions (4.7; 1.3–21.0), and cabbage (4.0; 1.1–15.9). When analysis was limited to the 16 confirmed case-patients and their corresponding 48 controls, only green onions remained significantly associated with illness (17.6; 2.5–775.7). Restaurant invoices from chain A were collected for all items identified during the epidemiologic investigation, but efforts to trace any food item to its source were inconclusive. Although the current study identified potential foods associated with illness in Texas, investigators were not able to identify the illness source or confirm whether the patients within the chain A subcluster had consumed a product reported by other ill persons in the United States.

Cyclosporiasis is an intestinal illness caused by the parasite *Cyclospora cayetanensis*. Since 2013, the United States has experienced annual increases in the incidence of cyclosporiasis incidence during the summer months, with some illnesses linked to imported produce (*1*–*3*). Molecular subtyping of *Cyclospora* is not currently available; therefore, identification of an ingredient associated with a particular illness subcluster might provide information about a source contributing to other cyclosporiasis illnesses. Previous U.S. outbreaks of cyclosporiasis have been linked to fresh produce, such as prepackaged salad mix, raspberries, and cilantro (*3*,*4*). Identification of a vehicle for *Cyclospora* is complicated by the short shelf life of fresh produce as well as the use of potential vehicles such as garnishes or mixtures with other items that could also harbor the parasite. Ingredient-level analysis within restaurant clusters and subclusters therefore remains critical in *Cyclospora* outbreak investigations.
